# Resolutions of Respect

**Published:** 1891-02

**Authors:** 


### RESOLUTIONS OF RESPECT.

---

At a called meeting of the Atlanta Society of Medicine, Wednesday evening, January 21st, the following resolutions were unanimously adopted on the death of Dr. Joye, which occurred on the night of January 20th.

WHEREAS, It has pleased an all-wise Providence to take from our midst our beloved comrade, Dr. Edmund V. Joye, be it

*Resolved*, 1st. That in the death of Dr. Joye the Atlanta Society of Medicine loses one of her most worthy and active members—a courtly gentleman and a skilled physician.

2d. That the president of the society appoint four members, who, together with a like number from the South Carolina Society, act as pallbearers in transferring the remains from the house to the train, and that the remaining members of the society accompany the body as an escort.

3d. That we extend to the family of the deceased our heartfelt sympathies in this their hour of deep grief.

4th. That these resolutions be placed upon the minutes of the society, and that a copy of them be sent to the family of the deceased and the daily papers of this city.

### REPORT ON PROGRESS IN THERAPEUTICS.

BY FRANCIS H. WILLIAMS, M. D.

### SACCHARIN AS A MEANS OF ACIDIFYING THE URINE.

While it is usually not difficult to render an acid urine alkaline, our means of rendering an alkaline urine acid are less satisfactory.

When saccharin was first announced, it was mentioned among its properties that it was unaffected by the digestive fluids and was eliminated unchanged in the urine. Some time later, Dr. Andrew S. Smith had occasion to manipulate with this substance and was struck with its strongly acid property; it occurred to him that so decided an acid, of such a stable composition as to resist decomposition in the system, and electing the kidneys as its means of exit from the body, would supply exactly the agent required for acidifying the urine.

The idea was tested on a boy suffering with transverse myelitis, whose urine, which required to be drawn with the catheter, was ammoniacal and very offensive. A few grains of saccharin, administered three times a day, promptly changed the reaction of the urine to acid, and did away completely with the offensive odor; not only so, but the irritation of the bladder became less, and the formation of pus was diminished.

Shortly after this Dr. Smith was in attendance upon a case of subacute meningitis in a child twenty months old. The urine, which dribbled constantly into the diaper, was alkaline, and its odor, though not ammoniacal, was peculiarly sickening. Every effort was made in the way of cleanliness, but the atmosphere about the bed was extremely disagreeable. Small doses of saccharin were prescribed, and immediately removed the fetor, to the great relief of the parents and attendants.

It is probable that a part of the efficacy of saccharin in these cases is due to its being a powerful antiseptic, in addition to its acid property.—*Boston Medical and Surgical Journal, November* 27, 1890.

---

### ALCOHOL AND ALCOHOLIC SOLUTIONS IN THE ABORTIVE TREATMENT OF HERPES.

In a thesis published by Dr. Dupas, of Lille, the following directions are given for the treatment of this common and often troublesome condition. Alcohol, ninety per cent. strength, or a solution of two parts resorcin to one hundred of alcohol, can be employed as a dressing; or one per cent. of thymol or three per cent. of menthol in ninety-five per cent. alcohol. If the solutions cause too much pain, a little cocaine may be added. Compresses moistened in one of the solutions are to be applied upon the lesions and over this spread some impermeable material, or absorbent cotton may be used. These dressings must be changed frequently during the day. The herpetic eruption aborts rapidly under this treatment. The element of pain is also subdued, and it is not rare to see rebellious neuralgia from herpes zoster give way in a few hours to this treatment.—*Boston Medical and Surgical Journal, November* 27, 1890.

---

STEIN. "The combination of resorcine and cocaine in the treatment of diseases of the ear."—*Revue de Laryngol., d'Otolog., etc.*, August, 1890.

The author has made an extended trial of resorcine in his practice, owing to its possessing slight astringent and caustic properties, well tolerated by the mucous membrane of the tympanic cavity. He has at the same time found that it possesses the property of favoring epithelial regeneration without at the same time causing any maceration of the tissues. The action of the drug, however, appears to be very superficial. Cocaine, on the other hand, favors the re-absorption of pathological exudations. The author accordingly uses a solution containing 1 per cent. of resorcine and 2½ per cent. of cocaine, to which may be added, if there is severe pain, a small quantity of morphia. A small qnantity of this solution is poured into the meatus, and is allowed to remain *in situ* for five minutes. This solution has produced the best effects in those cases where the drum is intact, where foreign bodies have been removed from the meatus, in myringitis, in otitis media with hæmorrhage, in chronic catarrh of the middle ear with hyperæmia; in this last case injections are made into the middle ear through the Eustachian catheter. This application is also useful in purulent disease of the middle ear. It must not, however, be employed in those cases where diminution of the suppuration produced by the action of the medicinal agent brings about impaired hearing power. Its principal use is in those cases which are accompanied by hyperæmia. —*Medical Chronicle*, 1890.

---

Electropuncture in Chronic Hydrocele. Dr. W. Zuelzer. (*Internat. Centralbl. f. d. Physiol. und Patholog. der Harn-und Sexual-Organe*, Band ii., Heft 2.)

This method of treatment, from which so much has been expected, has gradually lost ground, owing to the fact that recurrences took place in the vast majority of cases in which it was employed. Among the author's cases permanent recovery was observed in only one instance, although the application was made twice. He thinks, however, that the method is applicable in cases where the patient is so greatly debilitated that irritant injections or the radical operation are contra-indicated. Electropuncture is preferable to simple puncture, in that it is free from any ill effects ; and this is due to the fact that the absorption of hydrocele fluid takes place slowly and gradually. The author employs two platinum needles which are coated with an insulating layer up to the point. Both are inserted, as far apart as possible, into the sac of the hydrocele, connected with a constant battery; a current having a strength of 3 or at the most 4 milliampères is passed through them. The sitting should not exceed 2 to 2½ minutes. During the application active vermicular contractions of the scrotum are observed. The pain is inconsiderable if the current is not passed through for too long a period. In many cases, after the removal of the needles and careful closure of the punctures, a gradual dimunition in the size of the tumor ensued. Occasionally œdema of the scrotum occurred during the first few hours, which rapidly disappeared. Within eight days the fluid has been entirely absorbed, and by the employment of proper bandages and a snugly-fitting suspensory its re-accumulation can be prevented for a long time—in some of Zuelzer's cases for as long as six months. One of the advantages of the method is the rapidity with which it excites absorption, the quantity of the urine being materially increased during the first twenty-four hours after the operation. In all the cases the inner surface of the sac was found covered with coagula as early as the first day, so as to be no longer transparent, and even when the fluid re-accumulated the characteristic translucency was never as marked as before.—*Journal of Cutaneous and Genito-Urinary Diseases, January*, 1891.

---

### THE ETHICS OF EXPERIMENTATION UPON LIVING ANIMALS.

---

By STEPHEN SMITH BURT, M. D.

---

Cruelty may be defined as the infliction of suffering without sufficient reason. To be humane is an expression of the highest type of manhood. And surely less inhumanity can be traced through the records of science, than can be found in the annals of either religion or philosophy. Who is more altruistic, more practically philanthropic, than a physician of the better class in his daily intercourse with suffering mankind? Yet these men are periodically assailed by well-meaning if not well-informed persons, and stigmatized as wantonly cruel for performing what in the majority of instances are but painless operations upon otherwise useless animals. An action is good, bad, or indifferent, in direct ratio to its consequences. Now, those best able to judge admit that vivisection furthers the progress of scientific investigation, and, therefore, concede its defensibility. At the same time, when merely a matter of idle curiosity it is held to be most reprehensible. Frequent repetition of painless experiments, however, finds its justification in the necessity for objective instruction. No person will deny that science has conferred incalculable good upon mankind. Nay, more, the burdens of the lower animals are daily being lifted by this modern offspring of the human intellect. Were it not possible to specify an individual discovery due to vivisection, nevertheless the fact would remain that the crucial test of all hypotheses is experimentation. And it was not until verification became the anchor of all research that knowledge ceased to drift hither and thither upon the treacherous sea of speculation. But one of the most important steps in the advancement of physiological inquiry, the discovery of the inhibitory function of the pneumogastric nerve by Weber, was the result of an experiment upon a living animal, and it could have been demonstrated in no other manner. Moreover, this same method, in the hands of Pasteur, has be stowed sufficient benefit upon the animals themselves to more than compensate for the suffering inflicted upon them in the interest of science. Unbridled Nature is the personification of cruelty, and the few victims of scientific investigation that are sacrificed for the welfare of man are as nothing compared to the multitude that meet with apparently purposeless destruction. Certain facts are beyond the reach of the physiologist, except through these experiments; but since, in many instances, pain is a disturbing element, its abolition may be relied upon from this, if from no higher motive. The highest order of man feels unwilling to inflict needless suffering upon any living creature. How much right we have to the lives of the lower animals remains an open question. And though, at this stage of our civilization, it seems to be the general opinion, at least as expressed by man, that the animal shall be slain for our needs, still the causing of unnecessary pain is justly reprobated. Nearly all vivisections, assuredly in our country, are performed after the animals have been rendered insensible; but where sentiment blindly forms convictions, reason appears to hold no sway, hence, despite the facts of the case, these perennial crusades against a very necessary method of experimentation. Ultra-humanitarianism in its opposition to scientific advancement is liable to lapse into insipid sentimentality.—*The Post-Graduate, January*, 1891.

